# Nanoparticles of natural product-derived medicines: Beyond the pandemic

**DOI:** 10.1016/j.heliyon.2025.e42739

**Published:** 2025-02-19

**Authors:** Yedi Herdiana

**Affiliations:** Department of Pharmaceutics and Pharmaceutical Technology, Faculty of Pharmacy, Universitas Padjadjaran, Sumedang, 45363, Indonesia

**Keywords:** Coronavirus, Herbal medicine, Plant extract, Pandemics, Nanoparticles

## Abstract

This review explores the synergistic potential of natural products and nanotechnology for viral infections, highlighting key antiviral, immunomodulatory, and antioxidant properties to combat pandemics caused by highly infectious viruses. These pandemics often result in severe public health crises, particularly affecting vulnerable populations due to respiratory complications and increased mortality rates. A cytokine storm is initiated when an overload of pro-inflammatory cytokines and chemokines is released, leading to a systemic inflammatory response. Viral mutations and the limited availability of effective drugs, vaccines, and therapies contribute to the continuous transmission of the virus. The coronavirus disease-19 (COVID-19) pandemic has sparked renewed interest in natural product-derived antivirals. The efficacy of traditional medicines against pandemic viral infections is examined. Their antiviral, immunomodulatory, anti-inflammatory, and antioxidant properties are highlighted. This review discusses how nanotechnology enhances the efficacy of herbal medicines in combating viral infections.

## Graphical abstract

**Figure:**
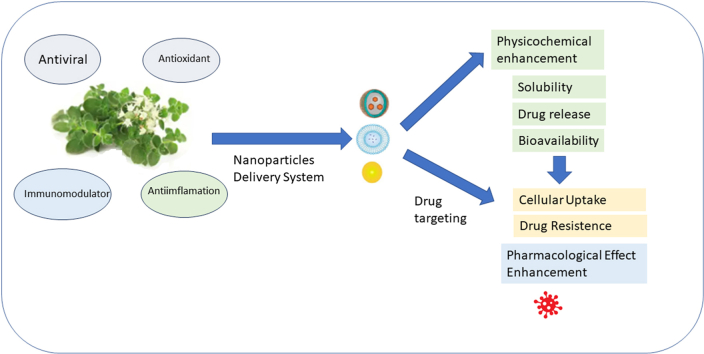

## 1 Introduction

Pandemics caused by highly contagious viruses profoundly impact global health and economies, creating an urgent need for solutions [[1], [2], [3], [4], [5]]. Antivirals and vaccines are essential to prevent viral infections and reduce mortality rates [6,7]. Testing and identifying infected individuals pose substantial challenges [8,9], while the emergence of viral variants can diminish the efficacy of antiviral therapies, thus emphasizing the need for preventive vaccines [10,11]. As society strives to regain normalcy, safe alternatives, such as natural products and nanotechnology, are essential to lessen the impact of the disease and overcome the limitations of isolation measures [12,13].

Immune dysregulation observed in severe cases of such pandemics, particularly among older individuals and those with compromised immune systems, contributes to more severe outcomes [14,15]. Severe-acute-respiratory-syndrome-related coronavirus-2 (SARS-CoV-2) impairs the immune system, triggering a harmful inflammatory response that hinders the body's defense [[15], [16], [17], [18]]. Common symptoms include fever, cough, difficulty breathing, lung damage, and fatigue [[19], [20], [21]]. Some individuals experience mild or no symptoms [[22], [23], [24]]. Severe cases can lead to respiratory failure, heart problems, and secondary infections [[25], [26], [27], [28]]. Underlying conditions like diabetes, heart disease, and kidney disease increase the risk of severe illness [29]. COVID-19 also affects the brain, causing symptoms like memory loss and loss of smell [30].

Conventional strategies focus on isolation, contact tracing, optimal care, early diagnosis, and the development of diagnostic tools, preventive measures, and vaccines [[31], [32], [33], [34]]. Supportive care forms the treatment basis, supplemented by broad-spectrum antibiotics, antivirals, corticosteroids, and convalescent plasma [32,[35], [36], [37]]. Clinical trials have investigated various drugs, including repurposed flu treatments, antivirals, and potential antibody therapies [[38], [39], [40]]. Vaccines have dramatically reduced deaths worldwide [41], but unequal distribution has limited their impact in poorer countries. Booster shots protect against COVID-19, especially among young people [[42], [43], [44]]. Researchers are exploring natural remedies to boost immunity, but effective treatments remain elusive [[43], [44], [45]].

Traditional remedies, particularly natural compounds derived from plants and animals, are being explored as adjuvant treatments for pandemic viral infections due to their potential antiviral and immunomodulatory properties [[46], [47], [48], [49]]. Products such as lemon, green tea, saffron, *Curcuma longa*, and various Indigenous medicinal plants have demonstrated antiviral and immunomodulatory activities [[50], [51], [52], [53], [54]]. These natural compounds are attractive due to their availability, affordability, and historical use in disease prevention [[53], [54], [55]]. These natural treatments are appealing because they are widely available, affordable, and have a long history of use. As we face ongoing pandemics, there's renewed interest in exploring these traditional approaches. This review examines how effective they are against viral infections.

Nanoparticles (NPs) enhance the efficacy of both traditional and modern medicines by improving drug delivery, solubility, and stability [56,57]. This integration could revolutionize herbal medicine, especially in the context of viral infections. Natural products, which have long been a source of therapeutic agents, offer significant promise due to their diverse pharmacological properties [58,59]. When combined with nanotechnology, these natural compounds can be further optimized. Nanotechnology improves bioavailability, stability, and targeted delivery, enabling more effective treatments [60]. Recent scientific advances highlight the potential of these enhanced herbal formulations to combat viral infections, including coronavirus disease, positioning them as promising candidates for future therapeutic strategies.

## 2 General overview of pandemic viral diseases

### 2.1 Viral diseases tend to pandemic

Viral diseases, such as the Black Death in the 14th century to the Spanish Flu in 1918, and more recently, the emergence of HIV/AIDS, Severe Acute Respiratory Syndrome (SARS), H1N1 influenza, Ebola, Zika, and the COVID-19 pandemic, have escalated to pandemic proportions, causing significant social, economic, and political disruption [[61], [62], [63]]. These events serve as stark reminders of our vulnerability to infectious diseases. Some of the deadliest diseases to stalk humankind have come from pathogens that jumped from animals to people [64]. Globalization, increased travel, and environmental changes like deforestation and climate change have created conducive conditions for the spillover of zoonotic diseases from animals to humans, leading to the worldwide spread of these diseases [[65], [66], [67]]. Under certain conditions, a newly emerged pathogen can escalate from an outbreak to a pandemic [68].

The COVID-19 pandemic has shown the importance of investing in healthcare infrastructure. In response to these evolving challenges, there is an immediate need for robust public health infrastructure [69]. Effective pandemic detection and monitoring depends on a strong public health data infrastructure and the collection and reporting of high-quality data [70]. Surveillance systems, early warning systems, and rapid response capabilities are critical for the timely detection, monitoring, and mitigation of infectious disease threats. Strengthening healthcare systems, promoting scientific research, and encouraging international cooperation are essential components of effective pandemic preparedness and response [68,71]. Coupled with this is the growing threat of antimicrobial resistance, spurred by the overuse and misuse of antibiotics and other antimicrobial drugs. This scenario further complicates disease management by fostering the development of drug-resistant strains. Overuse of antibiotics is accelerating antimicrobial resistance among pathogenic microbes, which is a growing public health challenge at the global level [[72], [73], [74]]. Antimicrobial resistance poses an increasing threat to global health, making developing new therapeutic strategies essential. Nanotechnology and natural products, including herbal remedies, offer promising alternatives to traditional antibiotics by targeting bacterial resistance mechanisms and enhancing the efficacy of existing treatments [75,76].

### 2.2 Origin and transmission of coronavirus

The origin and transmission of coronaviruses, particularly SARS-CoV-2, have been extensively studied. Genomic analysis shows that SARS-CoV-2 relates to SARS-like bat viruses, suggesting bats as a possible primary reservoir [77]. The two main hypotheses are natural zoonotic spillover, likely at the Huanan Seafood Wholesale Market, and a laboratory leak from the Wuhan Institute of Virology (WIV) [78].

Other viruses in the same phylogenetic group have caused previous outbreaks, including Severe Acute Respiratory Syndrome (SARS) and Middle East Respiratory Syndrome (MERS) [79]. Like these viruses, SARS-CoV-2 has a zoonotic origin. To reduce future pandemic threats, stricter regulation of wet markets and enforcement of wildlife trade laws are essential. Land-management efforts, such as halting deforestation, can create a buffer between wildlife and humans. Sustainable and humane farming practices can prevent overcrowding of domesticated animals and reduce prophylactic antimicrobial use, which also helps prevent antimicrobial resistance. Regardless of the virus's origins, these measures can mitigate future pandemic risks [78].

### 2.3 Human-to-human transmission routes

The intermediate source of SARS-CoV-2 and its transfer to humans is unknown, but rapid human-to-human transmission is well-documented [77]. SARS-CoV-2 infects human cells by recognizing the angiotensin-converting enzyme 2 (ACE2) receptor. This affinity is due to six amino acid residues in the Spike protein's receptor-binding domain (RBD) variable loop [80].

Human-to-human transmission is the primary mode of spread of SARS-CoV-2, the virus responsible for COVID-19. Key transmission routes include respiratory droplets, direct contact, and contaminated surfaces. Respiratory droplets from coughing, sneezing, or talking are the primary source, as nearby individuals can inhale them. Close contact, such as touching or shaking hands, can also facilitate transmission if the virus is transferred to the face, allowing entry through the eyes, nose, or mouth. Contaminated surfaces pose another risk; the virus can survive on surfaces for up to 9 days. Touching these surfaces and the face without proper hand hygiene can lead to infection. Asymptomatic individuals can transmit the virus, making preventive measures crucial even without symptoms [[81], [82], [83]].

Ongoing research explores additional transmission modes. Fecal-oral transmission is suggested by the virus's presence in stool samples and gastrointestinal tissues, highlighting the importance of proper handwashing [84,85]. Rare cases suggest the possibility of vertical transmission from mother to baby during pregnancy, childbirth, or breastfeeding, though more research is needed to understand this entirely [86].

Preventing the spread of SARS-CoV-2 requires adherence to public health guidelines, such as wearing masks, maintaining physical distance, frequent handwashing with soap and water, and regularly disinfecting commonly touched surfaces. While measures like social distancing and mask-wearing are broadly adopted, there is debate over the effectiveness of lockdowns and restrictions on movement [12,68]. By understanding the different transmission routes and implementing appropriate preventive measures, we can effectively curb the virus's spread and safeguard our communities from COVID-19 [87].

### 2.4 The end of the pandemic

The end of a pandemic is marked by significantly reduced disease spread. Several factors contribute to this: personal protective measures like handwashing and mask-wearing, developing immunity through vaccines or prior infection, effective antiviral treatments, and even natural changes in the virus or its environment [68,88,89]. However, it's important to note that the end of a pandemic doesn't mean the complete disappearance of a disease. Some diseases transition from pandemic to endemic status, meaning they continue to circulate within a population but at a manageable level. This is what happened with the H1N1 influenza virus [90]. The human H1N1 lineage caused pandemic and endemic influenza from 1918 to 1956, then disappeared entirely around 1957, and only reappeared in relatively low-level pandemic form in 1977. It has continued to circulate endemically in humans up to the present time. As of July 2024, COVID-19 remains a pandemic with ongoing global efforts to control its spread.

## 3 Current in the treatment of coronavirus

### 3.1 Current gaps in the treatment of coronavirus

Coronavirus infections remain a significant challenge for global health systems due to knowledge and resource gaps [91,92]. A critical need is for antiviral drugs to directly inhibit viral replication [90]. While protease inhibitors have been a research focus, a comprehensive understanding of the virus's interaction with the immune system is also crucial. COVID-19 has profound effects on the immune system, including the development of autoimmunity. To develop effective treatments, we must unravel the complex mechanisms of immune dysregulation specific to different individuals and disease stages [15].

Another major challenge is managing the severity of coronavirus infections, often compounded by a hyperinflammatory response known as a cytokine storm [15,93]. Recognizing the factors contributing to this variability, such as age, sex, comorbidities, and genetic predispositions, is crucial for effectively tailoring treatment strategies and improving outcomes across different patient demographics [[94], [95], [96]]. Mutations and emerging variants of SARS-CoV-2 lead to heightened transmissibility, increased rates of illness and death, and pose challenges by evading diagnostic tests. They also demonstrate reduced responsiveness to antiviral treatments and antibody therapies and have the potential to reinfect individuals who have recovered from the virus or been vaccinated [97].

Damaging SNPs in antimicrobial peptides hBD-2 and LL-37 weaken their structural stability and binding affinity to SARS-CoV-2. These findings highlight the role of specific genetic variations in COVID-19 susceptibility, paving the way for personalized diagnostics and treatment strategies [98]. The treatment of severe cases of coronavirus infections often necessitates specialized care, including respiratory and organ support for complications such as acute respiratory distress syndrome (ARDS). Yet, the limited availability of effective therapeutics for severe cases represents a significant gap in our treatment arsenal. We must develop therapies targeting the specific pathophysiological mechanisms contributing to severe disease and organ dysfunction [99,100]. Additionally, integrating traditional medicine, with its rich history and potential therapeutic benefits, into mainstream treatment approaches is another key area that requires attention. Rigorous scientific studies are needed to validate the efficacy, safety, and optimal use of herbal remedies and medicinal plants in treating coronavirus infections [[101], [102], [103]]. Lastly, addressing disparities in global access to effective treatments is critical, a significant barrier in our fight against the pandemic. Achieving global collaboration and equitable distribution of treatments is crucial to ensure access to life-saving interventions for all individuals, regardless of their socioeconomic background [104,105].

### 3.2 Anti-coronavirus therapies

Potential coronavirus treatments can target either the human body or the virus itself. One approach is to boost the immune system, particularly the innate response through interferons. Blocking cellular pathways used by the virus can also be effective. Directly targeting the virus involves preventing viral Ribonucleic Acid (RNA) synthesis, inhibiting key enzymes, blocking receptor binding, or disrupting viral assembly [106]. Many plant-based compounds, including flavonoids, alkaloids, and terpenoids, have shown antiviral, antioxidant, or anti-inflammatory properties through various mechanisms [107].

#### 3.2.1 Antiviral

Viruses are microscopic pathogens that rely on a host to replicate and survive [108]. Their ability to rapidly mutate and spread has made them a persistent threat to global health, causing a spectrum of diseases from mild to deadly [109]. Antiviral drugs have been instrumental in combating these infectious agents, designed to target either the virus or the host's response. However, the emergence of drug-resistant strains underscores the urgent need for continuous research and development [110].

Traditional medicine has offered potential antiviral solutions for centuries, with many plant-based compounds demonstrating antiviral properties. While these natural remedies can complement modern medicine, their efficacy varies, and rigorous scientific evaluation is essential [108]. The complex interplay between viruses and the human immune system necessitates a more profound understanding to inform the development of more effective antiviral strategies. Advances in genomics, immunology, and structural biology are providing new avenues for targeted antiviral therapies, including exploring immune-boosting approaches and designing antiviral agents that interfere with viral replication at multiple stages [110].

#### 3.2.2 Immunomodulator

Viral infection hinges on the complex interplay between viral and human proteins [111]. Viruses exploit host cell machinery for replication while simultaneously evading immune defenses. SARS-CoV-2, a prime example, uses its spike protein to bind to human ACE2 receptors, initiating infection. The virus produces proteins that interfere with the immune response, sometimes contributing to severe disease [97,112,113]. The human immune system mounts a multifaceted response to SARS-CoV-2, involving various immune cells [[114], [115], [116]]. However, an immune system is highly complex, and an overactive immune response can cause excessive inflammation and tissue damage. Of particular importance to immune resilience, individuals with weakened immune systems are especially susceptible to severe COVID-19 [[117], [118], [119]].

Traditional medicines influence the immune system in various ways. They can stimulate immune responses, suppress overactive inflammation, or balance both [120]. For example, Echinacea and Astragalus can boost immune function, while turmeric can reduce inflammation [121,122]. Essential oils and compounds like flavonoids also exhibit immunomodulatory properties via the utilization of their constituents, such as antibodies, cytokines, and dendritic cells. Some essential oils may stimulate the proliferation of immune-competent cells, including polymorphonuclear leukocytes, macrophages, dendritic cells, natural killer cells, and B and T lymphocytes [123]. These natural remedies have shown promise in managing viral infections, including COVID-19, but should not replace standard medical care [122]. Effective use requires understanding specific compounds, preparation methods, and individual responses. Ongoing research is essential to optimize their therapeutic potential.

#### 3.2.3 Anti-inflammatory

Inflammation is a prominent underlying factor in the development and pathogenesis of many chronic diseases. Globally, chronic diseases are the most significant causes of death, totaling 60 % of all deaths [124]. Inflammation-associated diseases encompass a range of infectious diseases and non-infectious inflammatory diseases, which continuously pose one of the most serious threats to human health, attributed to factors such as the emergence of new pathogens, increasing drug resistance, changes in living environments and lifestyles, and the aging population [125]. Inflammation in coronavirus infection, particularly with SARS-CoV-2, begins when the virus enters the body through the respiratory tract, binding to ACE2 receptors on host cells. This triggers the initial immune response where infected cells release cytokines and chemokines to attract immune cells like macrophages and neutrophils to the infection site [126,127]. These immune cells produce various cytokines, such as interleukins (e.g., IL-6), tumor necrosis factor-alpha (TNF-α), and interferons (IFNs), which coordinate the immune response and promote the clearance of the virus. The inflammatory response increases blood vessel permeability, allowing more immune cells to reach the infected tissues, leading to local swelling, redness, heat, and pain [128,129].

In severe COVID-19 cases, the immune response can become dysregulated, resulting in a cytokine storm and an excessive release of cytokines that causes widespread tissue damage. This overwhelming inflammation can lead to acute respiratory distress syndrome (ARDS), characterized by fluid buildup in the lungs' alveoli, causing severe breathing difficulties and reduced blood oxygen levels. The cytokine storm and systemic inflammation can also affect multiple organs, leading to multiorgan failure, a significant cause of mortality in severe COVID-19 patients [130,131]. Thus, while inflammation is crucial for fighting the virus, excessive inflammation can cause severe complications and death.

Herbal medicines have been given more and more attention for their remarkable curative effects and fewer side effects. Herbal medication can act in multiple pathways to prevent and treat rheumatoid arthritis through numerous components, including flavonoids, alkaloids, phenylpropanes, terpenes, etc. The main pharmacological effects are pain relief, inflammation improvement, immune function regulation, cartilage protection, pannus formation reduction, synovial hyperplasia inhibition, etc. [132]. The natural products of these plants demonstrated promising anti-inflammatory activities to treat skin, liver, cardiovascular, joint, gastrointestinal, neurological, and lung inflammation diseases [133].

#### 3.2.4 Antioxidants

Oxidative stress, an imbalance between oxidative products and antioxidants, plays a significant role in viral infections, including SARS-CoV-2 pathology. During COVID-19, increased oxidative stress can induce inflammatory damage and contribute to cytokine storms. This oxidative stress, coupled with inflammation, is associated with pulmonary injuries such as acute respiratory distress syndrome (ARDS). The rapid release of free radicals and cytokines leads to cellular injury, organ failure, and severe hypoxemia, which can critically harm the lungs and other organs [134,135]. Nitrosative stress promotes protein glycoxidation, and both processes can occur during infection with the SARS-CoV-2 virus [136].

Traditional medicines with antioxidant properties, such as curcumin and flavonoids in green tea, help neutralize reactive oxygen species (ROS), reducing oxidative stress and subsequent inflammation [[137], [138], [139]]. Flavonoids, including rutin, naringin, luteolin, caffeic acid phenethyl ester (CAPE), and quercetin, have shown potential as natural inhibitors of SARS-CoV-2. These compounds can prevent viral entry into host cells, decrease viral load, and reduce inflammatory reactions. *In vitro* and human studies have demonstrated their inhibitory effects on viral proteins and enzymes. Rutin, for example, has exhibited potent inhibitory effects comparable to remdesivir, a widely used antiviral drug. Similarly, luteolin, CAPE, and quercetin have shown efficacy against various viral targets [140,141]. These flavonoids possess potent anti-inflammatory properties, reduce oxidative stress, and protect against tissue damage, making them promising natural antioxidants in the fight against coronavirus infection.

## 4 Traditional herbal remedies for treating coronavirus infections

**Fig. 1:**
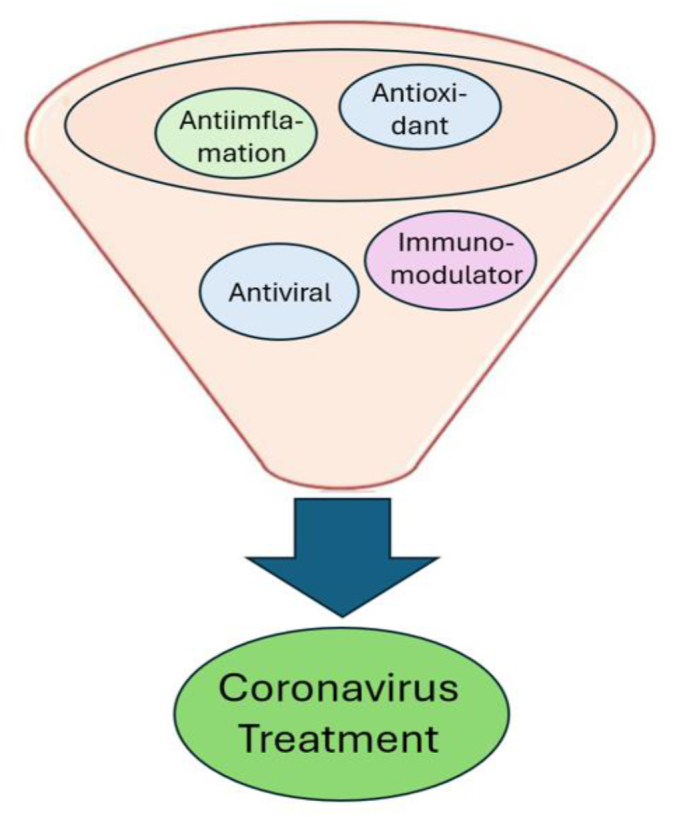
Potency herbal medicine.

The COVID-19 pandemic has increased interest in traditional herbal remedies [[142], [143], [144]]. Traditional medicines provide an invaluable supplement to our arsenal against viral infections, given their anti-inflammatory, anti-allergy, and antioxidant capabilities. The combination is expected to be a strength in fighting the coronavirus, as illustrated in Fig. 1. The multi-component and multi-functional capabilities are advantageous over conventional drugs [[145], [146], [147]]. Extracts of *Andrographis paniculata* and *Echinacea purpurea* exhibit antiviral activity against viruses like influenza, herpes simplex, and HIV or respiratory syncytial virus [148,149]. Beyond antiviral effects, these medicinal plants offer immunomodulatory and anti-inflammatory benefits with fewer side effects than conventional drugs [150].

Herbal medicines offer significant therapeutic potential due to their complex chemical compositions, but their efficacy and safety vary, requiring careful use and professional guidance. During the COVID-19 pandemic, research has examined their role in infection therapy, with some countries incorporating herbal remedies into treatment protocols under strict monitoring [151,152]. Numerous studies have been conducted to elucidate the pharmacological activity and molecular mechanisms of active compounds in herbal medicines, employing various methods, including in silico, *in vitro*, and *in vivo* analyses. *In silico* studies utilize computational models to predict molecular interactions and potential therapeutic effects, offering preliminary insights into how compounds might act at the molecular level. *In vitro* research involves testing these compounds in controlled laboratory settings, such as cell cultures, to observe their biological activity and mechanisms of action. Complementing these, *in vivo* studies evaluate the effects of the compounds within living organisms, providing critical data on their efficacy, safety, and physiological impact. Together, these approaches offer a comprehensive understanding of herbal medicines' therapeutic potential and inform their development and application. Table 1 illustrates the potential of herbal medicine, highlighting a single plant that contains various compounds with multiple effects. Numerous studies have substantiated these effects.

**Table 1:** Summary of active compounds in traditional herbal remedies with antiviral properties.

Herbal	Bioactive substance	Antiviral	Anti-inflammatory	Immunomodulator	Antioxidants	Ref.
Garlic (*Allium sativum*)	Allicin, diallyl sulfide (DAS), Z-ajoene	*A. sativum* exhibits antiviral activity, defending against HIV by inhibiting virus adhesion to host cells. It also has antiviral effects against SARS-CoV-2, HSV-1, HSV-2, and other viruses.	Garlic and its organosulfur compounds exert anti-inflammatory effects by modulating the NF-*κ*B pathway.	*A. sativum* helps maintain immune homeostasis and supports immune cells by regulating proliferation and cytokine gene expression.	Garlic contains numerous compounds, including vitamins B1, B2, B6, A, and C, and many antioxidants, flavonoids, phenolic compounds, and sulfur compounds.	[[153], [154], [155], [156]]
Ginger (*Zingiber officinale*)	Gingerol, polyphenols, shogaols, paradols, and zingerone	*Z. officinale rhizome* extracts have high potential for treating Chikungunya and exhibit antiviral action against SARS-CoV-2 by binding to proteases, Spike protein, RNA binding protein, and the N-terminal RNA-binding domain.	Its anti-inflammatory mechanism involves Akt inhibition and NF-κB activation, increasing anti-inflammatory cytokines and reducing pro-inflammatory cytokines.	In many studies, the immunomodulatory activities of gingerols, especially 6-gingerol, have been elucidated.	Ginger's antioxidant effects are linked to Nrf2 pathway activation, which reduces ROS formation and lipid peroxidation. It also lowers malondialdehyde (MDA) levels in stressed rats and ROS in fibrosarcoma cells.	[30,[157], [158], [159]]
Turmeric *(Curcuma longa* L.)	Curcuminoids and curcumin (CUR).	Curcumin inhibits viral replication by suppressing negative-strand RNA synthesis [158]. It has broad antiviral effects against HIV, hepatitis C and B, herpes simplex, and coronavirus [36]. Curcumin's mechanisms include virus attachment, entry, replication, and direct killing.	Curcumin reduces proinflammatory cytokines IL-6, TNF-α, and IL-17. Curcuminoids may mitigate COVID-19's effects, especially in the central nervous system, by inhibiting MAPKs, JNK, and NF-κB.	The curcuminoid extract of turmeric rhizome in VCO may have immunomodulatory activity.	Curcumin significantly reduced TMPRSS2 and TMPRSS11D expression, mitigating ROS levels induced by SARS-CoV-2. Curcuminoids inhibit carcinogenic ROS, including superoxide anions and hydroxyl radicals.	[30,160,161].
Black Cumin (*Nigella sativa* L.)	Thymoquinone, carvacrol ρ-cymene, trans-anethole, longifoline, and 4-terpineol.	*Nigella sativa* can reduce COVID-19 viral load, though it doesn't significantly affect hospitalization or recovery rates. Combined with methylene blue-based photodynamic treatment, it shows antibacterial and antiviral effects in wound care.	*Nigella sativa* oil has anti-inflammatory and antioxidant properties, affecting interleukins, tumor necrosis factors, and enzymes, suggesting benefits for inflammation-related conditions.	*Nigella sativa* significantly impacts the immune system, acting as a regulator by stimulating or suppressing immune responses. Thymoquinone, a key compound in N. sativa, influences T and B cells and interacts with inflammation-related signaling pathways.	The study results show that thymoquinone, trans-anethole, carvacrol, and 4-terpineol have antioxidant potential.	[[162], [163], [164]]
*Piper nigrum* L. (Black Pepper)	Piperine, oleoresin, essential oil, starch, and fatty acids.	Black pepper has antiviral properties and is effective against the parainfluenza virus. Piperine, a compound in black pepper, can inhibit SARS-CoV-2 entry into human cells.	Piperine, found in black pepper, has powerful anti-inflammatory effects. It reduces inflammation by regulating molecules like IL-1β and TNF-α and increasing anti-inflammatory molecules like IL-10 and TGF-β1. Piperine's influence on cellular pathways suggests its potential for treating conditions like sciatica.	Piperine acts as a potent immunomodulator. A study using human peripheral blood mononuclear cells (PBMCs) revealed that piperine significantly suppressed PBMC proliferation and the expression of IL-2 and INF-γ.	The essential oils of *P. nigrum* L. have strong antioxidant properties, reducing free radical damage. Piperine from white pepper shows some antioxidant activity, but the high activity of white pepper extract is likely due to interactions between piperine and other compounds.	[[165], [166], [167]]
*Tinospora cordifolia* (Willd.)	B-sitosterol, clerodane furano diterpene, columbin, tinosporine, tinosporide, tinosporaside, cordifolide, cordifol, heptacosanol, and furano diterpene.	*T. cordifolia* extract shows potential against HIV by stimulating immune cells and increasing hemoglobin levels. Additionally, its aqueous extracts influence cytokine production and activate effector cells.	The *in vitro* evaluation revealed significant anti-inflammatory activity of the *Tinospora cordifolia* stem aqueous extract.	*In vivo* studies show that Tinospora cordifolia extracts boost antibody production. *T. cordifolia* may combat coronavirus by enhancing the immune system and targeting specific genes and pathways, as evaluated through network pharmacology and *in vivo* studies.	*Tinospora cordifolia* exhibits potent antioxidant and free radical-scavenging properties, increases GSH levels, and boosts gene expression related to antioxidant activity. It also prevents lipid damage, neutralizes carcinogens, and reduces free radical generation and lipid peroxidation.	[[168], [169], [170]].

The integration of the multifunctional pharmacological properties of herbal medicine will have a positive impact on the treatment of coronavirus disease. Ginger, a potent antiviral, anti-inflammatory, and antioxidant remedy, is a potential therapeutic option for COVID-19 [171]. Understanding the molecular mechanisms of herbal medicines in COVID-19 treatment is essential.

However, translating the results and evidence from in silico, *in vitro*, and *in vivo* studies to clinical applications remains challenging due to critical limitations. Variability in experimental protocols—such as differences in dosing regimens, administration routes, and model organisms—compromises cross-study comparability and reproducibility. Interspecies differences in metabolism, immune response, and genetic pathways further hinder the extrapolation of *in vivo* findings to humans [[172], [173], [174]]. Additionally, preclinical models often overlook key human-specific factors, including genetic heterogeneity, comorbidities, and polypharmacy [[175], [176], [177]]. Addressing these barriers requires rigorous Phase I–III clinical trials, standardized experimental protocols, and integration of multi-omics approaches to uncover mechanistic insights and identify responsive patient populations [178,179]. Collaborative efforts involving computational biology, translational medicine, and regulatory science are essential to advance these interventions from bench to bedside.

## 5 Nanotechnology-enhanced herbal medicines for coronavirus treatment

**Fig. 2:**
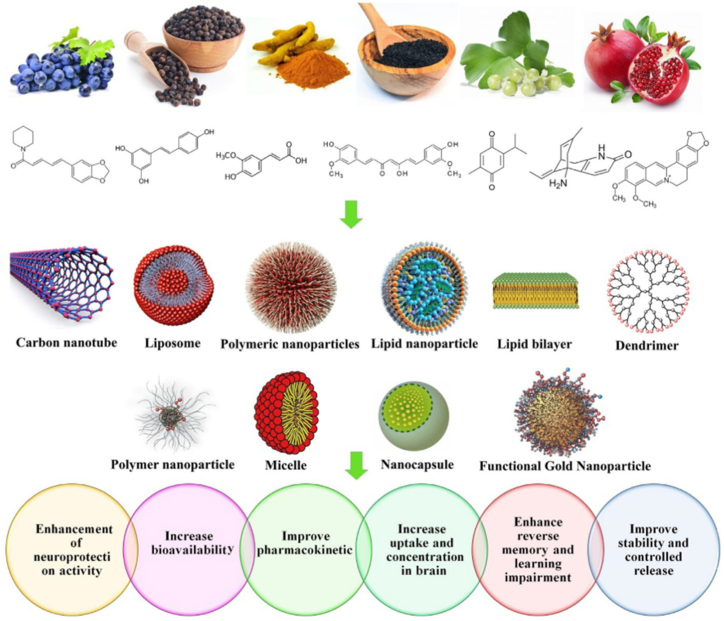
Nanoformulations used to improve the effectiveness of natural compounds [Reproduced [182] with kind permission from the copyright holder CC-BY – 4.0.].

No specific licensed therapeutic agents exist for COVID-19, with available treatments mainly supportive and not effectively halting infection or eliminating the virus. No particular drug combination has consistently been associated with mortality [180]. New, efficient antiviral drugs are urgently needed to reduce COVID-19 mortality and morbidity. Bioactive compounds from natural products and essential oils show strong potential as novel antiviral drugs. They reveal new structure-activity relationships and help develop effective therapeutic strategies. Although research is still preliminary, further studies are needed to characterize bioactive constituents, define mechanisms, and evaluate efficacy *in vivo*. However, the absence of comprehensive data on the safety and mechanisms of natural products limits their inclusion in clinical trials [120]. Developing combination therapies, such as polyherbal nanoformulations, as shown in Fig. 2, may reduce the risk of drug-resistant viruses. Phytopharmaceuticals will be crucial in creating safe and cost-effective nano-formulation delivery systems [181].

Pharmaceutical scientists are now focusing on nanocarrier-based drug delivery systems for herbal medicines. Therapeutic herbal or phytoproducts can easily dissolve in nonpolar solvents but are poorly soluble in water, resulting in partial absorption [183]. Nanostructured carrier systems such as polymeric NPs, liposomes, SLNs, polymeric micelles, and nanoemulsions have been investigated for delivering anticancer drugs orally, enhancing bioavailability, and reducing toxicities [184]. Nanomedicines employing different carrier materials have effectively resolved conventional drugs' delivery, solubility, absorption, and cytotoxicity issues [183]. Table 2 demonstrates that nanoformulation enhances the therapeutic efficacy of the extract. This allows extracts with multitarget therapies to offer better efficacy than single-purified compounds derived from the extract. Furthermore, nanoformulation can improve the bioavailability, stability, and targeted delivery of the therapeutic agents within the extract, leading to more efficient and controlled release profiles, reduced side effects, and potentially higher patient compliance. This underscores the potential of nano-formulated extracts in developing advanced therapeutic strategies.

**Table 2:** Nanoformulation strategies for herbal drugs.

Plant Extract	Formulation	Applications	Result	Ref.
Ethanolic extract of *C. longa* was pre-pared through Soxhlet extraction.	The nanosuspensions were formulated using four different stabilizers: sodium lauryl sulfate (SLS), hydroxypropyl methylcellulose, poly (vinyl alcohol) (PVA), and poly-sorbate-80.	The nanosuspension showed a reasonable dissolving rate, with maximum dissolution at pH 7, making it the most suitable condition for *C. longa* absorption in various solvents, mediums, or body fluids.	The study findings suggest that the nanoprecipitation approach enhances the dissolution potential and biological activities of *C. longa* root extract, resulting in significantly higher antioxidant and antimicrobial capabilities.	[185]
*S. marianum*, *E. cardamomum* and *C. sativum*	With slight modifications, the nano-precipitation method was used to prepare nanosuspensions with 1.5 % PVA.	Nanonization of herbal extracts can increase dissolution velocity, wetting, particle surface area, and saturation solubility.	Nanosuspensions of the selected plants *S. marianum*, *C. sativum*, and *E. cardamomum* significantly enhanced their antiradical potential compared to their crude extracts.	[186]
The ethanol extract of *C. cartilaginea*	Chitosan-Sodium tripolyphosphate	This enhancement was mainly due to the plant's active compounds and the nanoparticles' small size, boosting cellular bioavailability.	Chitosan nanoparticles demonstrated strong antigenotoxic properties, and their anticancer potential was significantly enhanced when loaded with C. cartilaginea extract.	[187]
Thyme essential oil (TEO) loaded in nanocochleates	Nano cochleates were made with Phospholipon G90, cholesterol, and calcium.	Essential oils have interesting functional properties such as antimicrobial and/or antioxidant.	Free TEO showed vigorous antioxidant activity (75.2 %) at 1 mg/ml concentration. Also, TEO-loaded NCs maintained this high scavenging activity (72.2 %), indicating that this innovative formulation was able to preserve the antioxidant activity of TEO constituents.	[188]
*Salix subserrata Bark* Extract-Loaded Chitosan NPs	Chitosan-Sodium tripolyphosphate	The stem bark was selected for nanoparticle formulation based on HPLC–PDA-ESI–MS/MS profiling and *in vitro* antioxidant assessment using free radical scavenging activity.	Chitosan NPs administration showed promising potent neuroprotective and antioxidative efficiencies against arsenic-induced oxidative threats.	[189]
Olive leaf extract (OLE) with chitosan NPs.	Chitosan-Sodium tripolyphosphate	When combined with NPs, olive leaf extract (OLE) is more effective in preventing cancer.	OLE-C-NPs kill cancer cells by causing them to die (apoptosis) or break down (necrosis). This suggests that OLE-CNPs could be used as an extra treatment for cancer.	[190]

Nanotechnology offers a powerful tool for transforming herbal medicines into more effective therapeutics. Nano formulations significantly improve drug delivery and therapeutic outcomes by addressing challenges like poor solubility and bioavailability. For instance, curcumin, a compound with limited bioavailability, becomes much more effective when encapsulated in nanocarriers. This technology is particularly promising in combating infectious diseases [125,191,192]. Nanomedicines enable targeted drug delivery, overcoming biological barriers and strengthening antiviral activity. Their potential is highlighted in the fight against emerging pathogens like SARS-CoV-2. For example, nano curcumin has shown promise in reducing inflammation and viral load in COVID-19 patients [192,193]. Curcumin-loaded lactoferrin NPs were developed to protect Sloan-Kettering Neuroblastoma Subclone (SK-N-SH) dopaminergic cells from rotenone-induced neurotoxicity, a model that mimics symptoms like Parkinson's Disease. Besides sustained retention, Cur's intracellular uptake and concentration increased, enhancing its neuroprotective effects [194]. Incorporating curcumin into nano curcumin form may be a viable method for overcoming its intrinsic limitations, and there are reasonable concerns regarding its toxicological safety once it enters biological pathways [195].

Recently, Asprea et al. developed and characterized innovative nanocarriers, the nano cochleate, to increase the stability and preserve the anti-oxidative properties of thyme essential oil [188]. Interestingly, research by Jahan et al. has shown that the nanoformulation of *Curcuma longa* extract retains its multiple effects, such as antibacterial and antioxidant activities [186]. This suggests the hypothesis that encapsulation of the extract can maintain these multi-effects and potentially enhance them compared to non-nano-formulated extracts. Recent developments in nanotechnology have further demonstrated that nanoencapsulation can improve solubility, protect bioactive compounds from degradation, and provide a sustained release, thereby maximizing the therapeutic potential of the extract.

The translation of nanomedicines from the lab level into marketed product faces several challenges, including characterization of physicochemical properties, pharmacodynamics, pharmacokinetics, process control, biocompatibility, nanotoxicity, scaling-up as well as reproducibility [[196], [197], [198]]. Regulatory barriers that hinder the rapid approval of new nanomedicines [199,200]. Addressing these concerns is essential for successfully translating nano-based therapies into clinical practice.

While nanotechnology presents exciting opportunities, challenges such as large-scale production, toxicity, and regulatory hurdles must be addressed for widespread clinical application. Future research should focus on developing multifunctional nanomaterials for combined diagnosis and treatment and optimizing formulations for specific diseases. The synergy between nanotechnology and herbal medicine holds great potential for improving human health and combating infectious diseases [191,201]. However, the absence of comprehensive data on the safety and mechanisms of natural products limits their inclusion in clinical trials [118]. To optimize this drug delivery system, a better understanding of different biological connection mechanisms and particle engineering is still needed [192].

Several NPs applications have demonstrated the ability to improve the stability and bioavailability of active compounds. For example, the encapsulation of curcumin (Cur) with soy isolate protein (SPI) and pectin (PE) significantly enhanced its photostability and thermal stability [202]. Additionally, as a hydrophobic model, thiolated dextrin NPs loaded with curcumin exhibited targeted release in the intestines *in vitro*, suggesting potential for improved bioavailability [203]. Quercetin-loaded niosomal NPs showed stability, as no significant changes in entrapment efficiency (EE) or particle size were observed after 30 days of cold storage, further supporting their suitability for long-term use [204]. Similarly, Zhao et al. (2024) demonstrated that encapsulating curcumin into NPs effectively improves bioavailability [205]. Furthermore, curcumin-loaded solid lipid nanoparticles (SLNs) have been shown to enhance curcumin's bioavailability and anticancer effects, highlighting the therapeutic potential of nanoparticle encapsulation [206]. These studies emphasize NPs' role in improving bioactive compounds' stability, release profiles, and therapeutic efficacy.

NPs enhance the stability, permeability, biodistribution, improved loading capacity, and controlled release of active compounds, including natural products [207,208]. For example, curcumin—the active compound in turmeric—is often used as a model because of its easily degraded properties by light, pH, and enzymes. The stability of curcumin can be enhanced by encapsulation in NPs, which provide physical protection from environmental factors and create a stable microenvironment to maintain its biological activity [208]. In addition, NPs prevent the aggregation of active compounds, which is often a problem for natural products with low solubility, such as curcumin. The fate and aggregation of NPs in the subsurface are essential due to potentially harmful impacts on the environment and human health [209].

In terms of permeability, NPs allow natural products such as quercetin (from onions) to more easily penetrate biological membranes. Smaller NPs can penetrate cells more efficiently, leading to higher intracellular natural product concentrations, such as PEGylation or the addition of specific ligands, increasing affinity for target membranes. Their nanoscale size allows them to effectively penetrate cell membranes and increase their stability, which enables the drug to stay in circulation longer [210]. The biodistribution of natural products such as resveratrol (from grapes) can also be enhanced using NPs. Polyphenol resveratrol (RSV) undergoes extensive phase II metabolism, which limits its bioavailability and bioactivity [211,212]. PEGylation of NPs also prolongs the circulation time of resveratrol, avoiding detection by the immune system and increasing the chance of reaching target tissues. Enhanced Permeability and Retention (EPR) effects help accumulation in target tissues, such as tumors, while active targeting using specific ligands, such as folate, allows direct delivery to cancer cells [213].

NPs allow for the controlled release of natural products, such as the gradual release of curcumin from a PLGA polymer matrix to maintain therapeutic concentrations for a more extended period [214]. In addition, pH-responsive release allows NPs to release quercetin in acidic environments, such as tumors or gastric tissues [215]. With this approach, NPs improve the stability and bioavailability of natural products and ensure targeted and efficient drug delivery. This technology makes NPs a superior platform for developing natural-based drugs with broad applications in pharmaceuticals, cosmetics, and modern medicine, as shown in Table 3 [216,217].

**Table 3:** Overview of various nanoformulations.

Nano-Formulation	Natural Products	Advantages	Limitations	Ref.
**Polymeric** **NPs**	Curcumin, Mangosteen	Controlled release, customizable surface properties, biodegradability. They are excellent for drug delivery due to their water solubility, biocompatibility, and storage stability.	Risks of particle aggregation and toxicity, complex synthesis process.	[218]
**Liposomes**	Curcumin, Resveratrol	Biocompatible, enhances solubility and stability, targeted delivery, reduced toxicity, and multidrug resistance.	High production cost, short half-life, fewer stable, leakage, and fusion encapsulated drug.	[219]
**Nanoemulsions**	*Eucalyptus staigeriana* essential oil, Garlic oil	Improved bioavailability, production ease, scalability, solubility, and physical stability.	Prone-to-phase separation requires surfactants and a higher cost of investment in equipment.	[220,221]
**Solid Lipid Nanoparticles (SLNs)**	Curcumin, Quercetin	High stability, controlled release, and protection against degradation.	Limited drug loading capacity, potential lipid polymorphism, and the potential for drug leakage.	[222]
**Dendrimers**	Epigallocatechin gallate (EGCG), Retinoicacid	Precise molecular architecture, high drug-loading efficiency, drug solubility, and stability in biological systems.	Not a suitable candidate carrier for hydrophilic drugs, Cellular toxicity, High cost of synthesis, and potential toxicity.	[223,224]
**Metallic** **NPs**	Flavonoids, Polyphenols	Antimicrobial properties, performance stability, processing simplicity, cytotoxicity.	Potential cytotoxicity, environmental concerns.	[225]

The combination of curcumin with natural products or vitamins continues to demonstrate promising therapeutic effects against COVID-19. A system biology analysis showed that combining curcumin, vitamin C, and glycyrrhizic acid could regulate immune and inflammatory responses associated with coronavirus infections [100]. A combination of ginger and garlic extracts displayed significantly higher (85.44 %, p < 0.005) antioxidant activity even at lower concentrations (6 mg/ml) compared to ginger and garlic extracts alone [159]. Another randomized controlled trial confirmed the superiority of this drug combination; oral supplementation with curcumin, quercetin, and vitamin D3 expedited SARS-CoV-2 RT-PCR test negativization, improved symptoms, reduced excessive inflammatory responses, and exhibited good tolerance and safety [160]. The Meniran and Turmeric combination could modulate the immune system, especially macrophage cell profiles, against cancer [162].

In parallel, natural-based drugs have attracted attention for their potential benefits in combating COVID-19. Using nanoformulation for herbs has advantages and disadvantages, as shown in Table 3. These natural compounds, rich in antioxidant properties and immune-boosting capabilities, have shown virucidal effects against several enveloped viruses. Preliminary studies suggest that these natural-based drugs could be supportive in managing COVID-19, although further clinical trials are necessary to substantiate these claims. The COVID-19 pandemic has highlighted the potential of natural products as effective anti-SARS-CoV-2 drugs [[226], [227], [228]].

## 6 Perspectives

Various drug delivery system applications via NPs have encountered an enormous position in sectors like pharmaceutical, medical, biological, and others [56]. Nanotechnology offers a promising avenue for addressing global health challenges, especially infectious diseases. Its applications span prevention, diagnosis, and treatment, exemplified by its pivotal role in the COVID-19 pandemic. NPs enhance the efficacy of both traditional and modern medicines. By improving drug delivery, solubility, and stability, nanotechnology can revolutionize herbal medicine, as highlighted by Teja et al. [[229], [230], [231]]. In infectious disease diagnosis, nanoparticle-based technologies enable rapid and sensitive detection of pathogens, facilitating timely interventions—for instance, lateral flow assays and nanopore sequencing offer efficient and cost-effective diagnostic solutions [232].

Moreover, nanotechnology has transformed vaccine development. As demonstrated in mRNA COVID-19 vaccines, lipid NPs have proven instrumental in achieving widespread immunization. This technology holds promise for addressing future viral outbreaks by enabling rapid vaccine production and adaptation [[233], [234], [235], [236]]. Nearly any antiviral drug delivery system can be nanoscale to the effect of improved properties [191,237,238]. The intersection of nanotechnology and infectious disease research offers a promising path forward in global health. Continued collaboration among scientists, policymakers, and healthcare professionals is essential to harness the full potential of this technology [239]. The COVID-19 pandemic highlighted the critical need for rapid development of antiviral medications, diagnostics, and vaccines. Nanotechnology has the potential to significantly enhance drug delivery and efficacy in combating these viral threats. Understanding the immune response to long COVID-19 is essential for developing effective treatments [240]. Nanoscale innovations have substantially advanced the potential of stem cell therapy by enhancing differentiation, proliferation, and therapeutic efficacy. Leveraging nanotechnology, stem cell therapy demonstrates significant promise in addressing pulmonary damage caused by SARS-CoV-2 infection by facilitating tissue regeneration and repair. Integrating nanomaterials enables precise modulation of stem cell behavior and function, offering innovative solutions to overcome existing therapeutic limitations [241].

Ginger extracts, mainly those rich in phenolics, flavonoids, and vitamin C, offer potential health benefits. Organic solvents excel at extracting these compounds, while water is more effective for vitamin C [159]. While traditional solvent extraction remains standard, advanced techniques like supercritical fluid extraction, pressurized liquid extraction, and ultrasound-assisted extraction offer improved efficiency and selectivity for extracting a broader range of metabolites. These modern methods often require specialized equipment but can yield higher quality extracts with minimal solvent residues—emerging techniques such as microwave-assisted and enzyme-assisted extraction show further promise for optimizing the extraction process.

Natural compounds like those found in ginger, combined with innovative technologies, hold promise for addressing viral threats. Silver NPs, derived from natural sources, exhibit antiviral properties against various viruses, though their mechanisms of action require further study [167,242]. Metabolomics, a field focused on small molecules in biological systems, has accelerated the discovery of new therapeutic agents from plants. However, identifying the specific compounds responsible for antiviral effects remains challenging [109].

While nanotechnology offers exciting possibilities for drug delivery and efficacy, challenges such as high production costs and potential toxicity persist. Balancing the benefits and drawbacks of nanotechnology while continuing to explore the potential of natural compounds is essential for developing effective and sustainable solutions to combat infectious diseases. Nanotechnology is rapidly becoming the next level of scientific technology. In vitro research has shown that encapsulating dietary supplements in NPs increases their delivery, stability, and availability [243].

The synergy between natural products and nanomaterials offers a promising strategy to combat drug resistance, a significant hurdle in contemporary therapeutics [[244], [245], [246]]. Natural products, including plant-derived compounds, microbial metabolites, and marine bioactives, exhibit various biological activities and inherent therapeutic potential. However, poor solubility, stability, and bioavailability often hinder their clinical translation. Nanomaterial-based delivery systems can effectively address these limitations, enhancing their therapeutic efficacy. By enabling controlled and targeted delivery, these systems improve drug accumulation at the disease site, minimizing off-target effects and mitigating the development of drug resistance [247,248].

Green nanotechnology offers a sustainable pathway to develop nanomaterials with minimal environmental impact. This approach emphasizes using environmentally friendly, renewable resources and energy-efficient methods for nanoparticle synthesis [[249], [250], [251]]. Green synthesis approaches, including plant-mediated synthesis, microbial fabrication, and agricultural or industrial waste product utilization, have attracted significant attention due to their low toxicity and cost-effectiveness [252]. These methods minimize dependence on hazardous chemicals and are in line with the principles of a circular economy by reusing waste materials [253,254]. These biological agents act as reducing and stabilizing agents, facilitating the formation of NPs under mild reaction conditions without toxic chemicals. For instance, metal NPs synthesized using plant extracts have demonstrated enhanced biocompatibility and reduced cytotoxicity, making them suitable for drug delivery applications [255,256]. Additionally, microbial synthesis using bacteria like *Bacillus subtilis* and fungi such as *Aspergillus niger* offers precise control over particle size and morphology [257,258].

Nanoparticle-based technologies face several significant challenges that hinder their widespread adoption and practical application. One major hurdle is ensuring the scalability and cost-effectiveness of the manufacturing process, as current methods often lack the economic and logistical feasibility required for large-scale implementation. Maintaining the stability of NPs during storage and transportation is another major hurdle. These systems are susceptible to external factors such as temperature fluctuations and are prone to aggregation, which can significantly compromise their effectiveness and reduce shelf life [198]. Regulatory challenges pose significant barriers to meeting stringent safety, efficacy, and quality control requirements in nanoparticle characterization [[259], [260], [261]].

A key step forward in personalized medicine is the development of multifunctional NPs. These engineered particles unite diagnosis and treatment as a single system known as theranostics [262,263]. NPs are multifunctional, delivering drugs directly to diseased cells to minimize side effects. Imaging or biomarker detection of these particles offers real-time information on drug distribution, treatment effectiveness, and disease progression [264,265]. Theranostics NPs combine diagnostic and therapeutic capabilities for more efficient disease management [262]. Multifunctional NPs offer significant advantages in treatment, delivering drugs directly to diseased cells and reducing unwanted side effects. Simultaneously, these particles provide valuable real-time information on drug distribution, treatment effectiveness, and disease progression by integrating diagnostic capabilities [266,267]. These NPs present a promising therapeutic avenue that requires future research efforts to improve their biocompatibility, scale up the production process, and meet current regulations to enable their clinical translation and widespread application.

New insights into nanoparticle-based drug development highlight new trends and innovative approaches transforming various fields. Advances in bioinspired nanoformulations, which mimic biological structures and processes, provide more efficient and biocompatible delivery systems [268,269]. Integrating artificial intelligence (AI) into formulation design will revolutionize and enable precise optimization of nanoparticle properties and simplify the drug development process [270,271]. AI is transforming nanotechnology by optimizing nanoparticle synthesis, process control, and quality assurance, enhancing efficiency and precision in manufacturing [272,273]. Predictive modeling enables the simulation of complex nanoscale phenomena, streamlining the design and development of advanced nanomaterials [274]. In pharmaceuticals, AI facilitates data-driven drug formulation, personalized medicine, precision targeting, and faster clinical trials [275]. Its integration improves product quality, reduces costs, and accelerates time-to-market, offering strategic insights for innovation and competitiveness.

Furthermore, nanotechnology plays a key role in enhancing the therapeutic potential of natural products by improving solubility, bioavailability, and targeted delivery. These advances open new avenues for creating more effective and accessible treatments, overcoming some limitations traditionally associated with natural product-based therapies. Integrating natural products with nanotechnology offers significant promise for combating viral infections, but several challenges remain, including safety concerns, scalability, and regulatory barriers. Further research is needed to explore the long-term safety of nanoformulations, optimize green nanotechnology methods, and address the regulatory complexities associated with the commercialization of these therapies.

## 7 Conclusion

Nanotechnology will play a pivotal role in preventing and managing future viral pandemics. Effective collaboration among scientists, policymakers, and healthcare professionals is essential to exploit its potential fully. Integrating nanotechnology with natural products offers significant promise in combating COVID-19 by enhancing therapeutic effectiveness, reducing side effects, and improving patient outcomes. Furthermore, traditional medicines, known for their antiviral and immunomodulatory properties, provide valuable resources for managing viral infections. Combining advanced technologies, natural products, and traditional medicines can strengthen public health resilience and better prepare us for future pandemics.

## Author contribution statement

This manuscript is solely authored by Yedi Herdiana. Yedi Herdiana was responsible for the conception and design of the study, data collection, analysis and interpretation of results, drafting the manuscript, and revising it critically for important intellectual content. The author has approved the final version of the manuscript for submission and agrees to be accountable for all aspects of the work.

## Additional information

No additional information is available for this paper.

## Data availability statement

Data included in the article is referenced in the article.

## Funding statement

This Article Processing Charge (APC) was funded by 10.13039/501100015690Universitas Padjadjaran.

## Declaration of competing interest

The authors declare that they have no known competing financial interests or personal relationships that could have appeared to influence the work reported in this paper.
